# Preparation and Characterization of Antimicrobial Films Based on LDPE/Ag Nanoparticles with Potential Uses in Food and Health Industries

**DOI:** 10.3390/nano8020060

**Published:** 2018-01-24

**Authors:** Dania Olmos, Gloria María Pontes-Quero, Angélica Corral, Gustavo González-Gaitano, Javier González-Benito

**Affiliations:** 1 Department of Materials Science and Engineering, Instituto de Química y Materiales Álvaro Alonso Barba (IQMAA), Universidad Carlos III de Madrid, Leganés 28911, Madrid, Spain; gpontes@ing.uc3m.es; 2 Department of Bioengineering and Aerospace Engineering, TERMeG, Universidad Carlos III de Madrid, Leganés 28911, Madrid, Spain; angelica.corral@uc3m.es; 3 Department of Chemistry, Facultad de Ciencias, Universidad de Navarra, Pamplona 31080, Spain; gaitano@unav.es

**Keywords:** polyethylene, silver nanoparticles, antimicrobial materials, high energy ball milling

## Abstract

In this work, the antimicrobial effect of silver nanoparticles in polyethylene based nanocomposites has been investigated using a non-conventional processing method to produce homogeneous materials. High energy ball milling under cryogenic conditions was used to achieve a powder of well-blended low-density polyethylene and commercial silver nanoparticles. The final composites in the form of films were obtained by hot pressing. The effect of various silver nanoparticles content (0, 0.5, 1 and 2 wt %) on the properties of low-density polyethylene and the antimicrobial effectiveness of the composite against DH5α *Escherichia coli* were studied. The presence of silver nanoparticles did not seem to affect the surface energy and thermal properties of the materials. Apart from the inhibition of bacterial growth, slight changes in the aspect ratio of the bacteria with the content of particles were observed, suggesting a direct relationship between the presence of silver nanoparticles and the proliferation of DH5α *E. coli* (*Escherichia coli*) cells. Results indicate that these materials may be used to commercially produce antimicrobial polymers with potential applications in the food and health industries.

## 1. Introduction

As food safety levels continue to become more sophisticated, the food industry is always looking for ways to improve food preservation and enhance quality control. Proper storage of food is essential for extending its shelf-life without losing nutrients, and for preventing and controlling foodborne infectious diseases [1,2]. Therefore, packaging materials play an important role in both the food and health sectors. Innovations in the packaging industry have focused on the development of novel active multifunctional materials to provide global solutions to food safety [3]. Unlike ordinary food containers, active packaging materials have been specifically designed to reduce permeability to oxygen or moisture, to protect the food from heat and light exposure, or even as sensors interacting with the food to provide information on potential deterioration or contamination.

Two examples of the most widely used active packaging are antimicrobial and controlled released packaging [4]. In controlled released packaging, an active substance incorporated in the packaging material, usually an antioxidant or a food preservative is gradually absorbed into the food to prevent its deterioration. On the other hand, antimicrobial packaging refers to the incorporation of antimicrobial substances in the packaging material. The aim in both cases is to prevent the growth of bacteria and biofilms on the food’s surface where the degradation process usually begins. This is a very important issue since even a few bacterial cells surviving in food are sufficient to cause illness [1].

Among the different packaging materials used in the food industry, plastics are the most commonly used due to their low cost and easy processability. In particular, polyethylene (PE) is one of the polymers most often used for a variety of applications because of its good corrosion resistance, low permeability to water, easy processing, and versatility. Research in this area has focused on the development of polymer nanocomposites with improved mechanical, thermal, or antimicrobial performance. Well-known examples of nanoparticles with antimicrobial properties include zinc oxide [5], titanium dioxide [5,6], and silver nanoparticles [7,8].

Silver has historically been used to eliminate bacteria and to avoid infections caused by bacteria. The antimicrobial mechanism of the silver comes from the generation of silver ions (Ag^+^) on the surface of the material when it comes in contact with water. Subsequently, these ions are transported by water to the bacteria, where the biocidal impact of the silver, known as the oligodynamic effect, causes their inactivation [9]. Another advantage of using silver nanoparticles is the enhanced activity due to the greater number of ions released as a result of the large surface areas associated with nanoparticles. Therefore, nanocomposites made from the mixture of silver nanoparticles (Ag NPs) and low-density polyethylene (LDPE) seem to be a good choice to prepare active packaging materials to be used as general-purpose containers, trays, plastic bags, tubing, wrapping films, etc. oriented to the food industry.

In polymer nanocomposite materials, optimum materials properties can be attained when there exists an efficient dispersion of the particles [10]. To date, researchers have explored a variety of processing techniques to achieve a uniform dispersion, including sol-gel, in-situ polymerization, and methods based on the chemical modification of the particles [11,12] or the polymer matrices [13,14]. Recently, high energy ball milling (HEBM) has been used to successfully prepare materials with a uniform particle dispersion [15,16,17,18,19,20,21]. In particular, HEBM has been used to prepare polymer nanocomposites for biodegradable polymer nanocomposites and electroactive polymer based materials [22]. In this work, HEBM under cryogenic conditions was used to disperse Ag NPs in a matrix of LDPE. Then, a subsequent hot-pressing step was used to prepare films of LDPE/Ag NPs.

Although LDPE is one of the most extensively used polymers for food packaging applications, there are just a few approaches for preparing antimicrobial packaging materials cost effectively. Except for the use of melt mixing [23,24] to prepare polyethylene based nanocomposites previous work found in the literature imply the use of more elaborate or longer protocols. Some examples of the methods used to prepare polyethylene/Ag nanocomposites are: (i) thermal reduction during melting [25,26]; (ii) supercritical fluid route [27]; (iii) in-situ polymerization [28,29]; (iv) casting [30,31]; (v) surface coating of LDPE using spraying or corona treatment [32,33] and (vi) layer-by-layer (LBL) deposition [34]. Therefore, the use of HEBM is proposed here as an alternative method, to prepare homogeneous LDPE/Ag nanocomposites with a uniform dispersion of the Ag NPs in the polymer bulk.

Our objective is to develop economically viable plastic materials resistant to the development of micro-organisms with potential applications in the food and health industries. In this work, we have focused on the preparation of LDPE based nanocomposites with different amounts of Ag NPs in order to study their resistance against bacterial growth and biofilm development for a strain of *E. coli* (*Escherichia coli*) (DH5α), and to better understand the direct effect of the presence of the Ag NPs on bacterial cell adhesion or biofilm development. To do this, a different approach is proposed to prepare the materials: the use of HEBM to disperse Ag NPs in polyethylene followed by a hot-pressing step to finally obtain films of the LDPE/Ag nanocomposites.

## 2. Results

### 2.1. Film Homogeneity and Nanoparticle Dispersion

**Figure 1:**
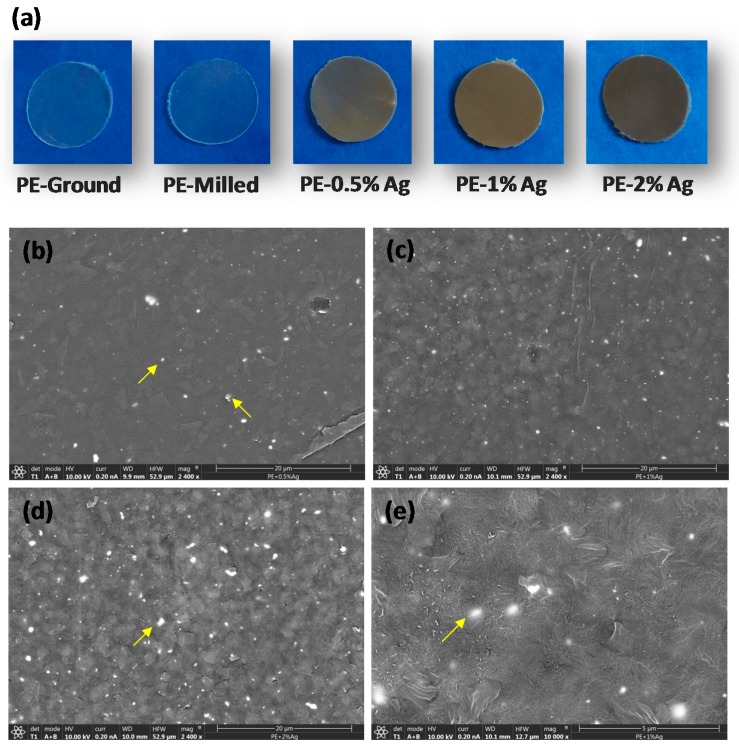
(**a**) Visual appearance of the samples cut in circular disks with a diameter of 1 cm; SEM micrographs obtained with BSE detector for: (**b**) PE-0.5% Ag; (**c**) PE-1% Ag; (**d**) PE-2% Ag and (**e**) magnification of (**c**) to show the size of the domains (where PE stands for polyethylene).

First, the overall homogeneity of the films was visually evaluated, as shown in Figure 1a. In general, the transparent materials were quite homogeneous, and the homogeneity did not change as a function of the amount of Ag NPs. For example, a brownish-grey color was observed for samples with Ag NPs, and the shade increased with Ag NP content. However, in the 0.5% Ag NP sample, some lighter regions were observed, indicating that the concentration of particles may vary slightly throughout the sample.

Second, scanning electron microscopy (SEM) of each sample surface was performed to assess particle dispersion in more detail. Figure 1b–e shows SEM micrographs obtained using the backscattered electron signal (BSE). Brighter regions of ~400–500 nm were identified and assigned to silver-rich domains. The analysis of particle-rich domains yielded an average size of approximately 340 nm. Considering the particle size of the commercial Ag nanoparticles (see Section 3: Materials and Methods), these domains were likely small aggregates of approximately 7–10 nanoparticles. The number of these domains increased with the content of Ag NPs in the film. Since the size of those brighter regions did not correspond to the size of a single particle, one can infer that HEBM did not effectively separate the Ag NP aggregates present in the ‘as received’ nanoparticles, though homogenous materials were obtained. The re-pressing process may also promote the formation of these aggregates (already present in the raw material) during film preparation.

### 2.2. Thermal Characterization

#### 2.2.1. Differential Scanning Calorimetry (DSC)

**Figure 2:**
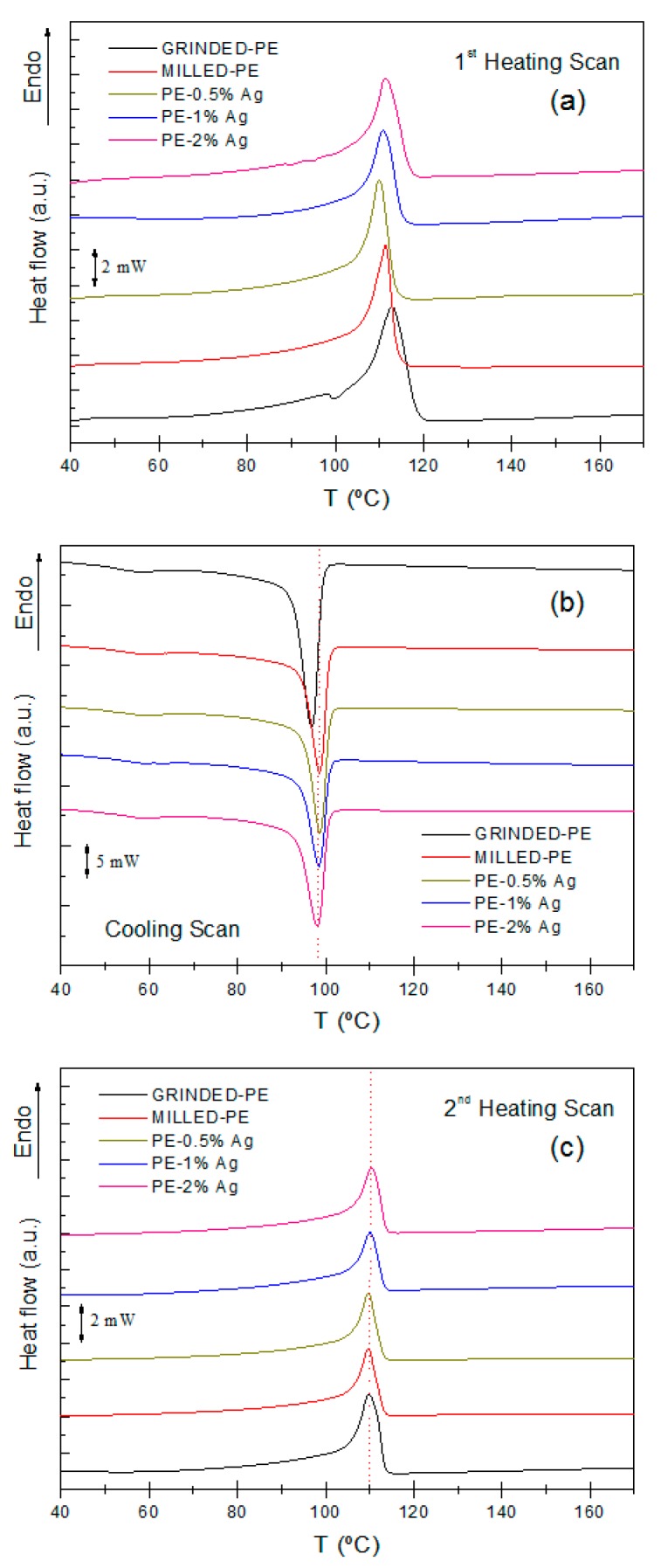
DSC traces corresponding to the first heating scan (**a**); cooling scan (**b**) and second heating scan (**c**).

In Figure 2, the DSC results corresponding to the first heating scan (Figure 2a), cooling (Figure 2b) and second heating scan (Figure 2c) are presented. In each case in the first heating scan at 20 °C/min, the materials exhibited a single melting peak, and the peak temperature varied slightly with Ag NP concentration (Figure 2a). These differences may be due to the processing conditions used. However, in the second heating scan (Figure 2c) these differences disappear, the peak temperature is located at approximately 110 °C, and the behavior during the melting process is very similar. Crystallization occurred at 98 °C (Figure 2b), in agreement with previous studies [6,35]. There is a secondary peak at ~60 °C which has been attributed to a thermal relaxation process [36], although its microscopic origin is not yet clear.

Table 1 summarizes the melting temperatures of the heating scans, the crystallization of the cooling scan, and the degree of crystallinity (*X*_c_). The degree of crystallinity was calculated by relating the melting enthalpy of the samples or the enthalpy of the cooling process to the melting enthalpy of a 100% crystalline sample of polyethylene, ∆*H*_m_^0^ = 289.9 J·g^−1^ using Equation (1) [37]:

(1) $$X_{c}=\frac{\frac{\left(\Delta H_{c}+\Delta H_{m}\right)}{2}}{\left(1-x\right)\cdot \Delta H_{m}^{0}}$$

where *X*_c_ is the degree of crystallinity, ∆*H*_c_ is the crystallization enthalpy, ∆*H*_m_ is the melting enthalpy and *x* is the number of particles present in the sample per unit mass (Raw data associated with the enthalpies of melting and crystallization can be found in the electronic supplementary material Table S1).

Melting and crystallization temperatures as well as the degree of crystallinity of the materials were similar to those of pure LDPE obtained in previous works [6,35,38], regardless of the Ag NP content. Therefore, the presence of these silver nanoparticles does not seem to affect the thermal properties of polyethylene, at least for the mass percentages used in this work. This is likely due to the lack of specific interactions between the Ag NPs and the LDPE chains, preventing the Ag NPs from acting as nucleating agents, which may induce changes in the sizes of the lamellae and/or spherulites [35].

#### 2.2.2. Thermogravimetric Analysis (TGA)

**Figure 3:**
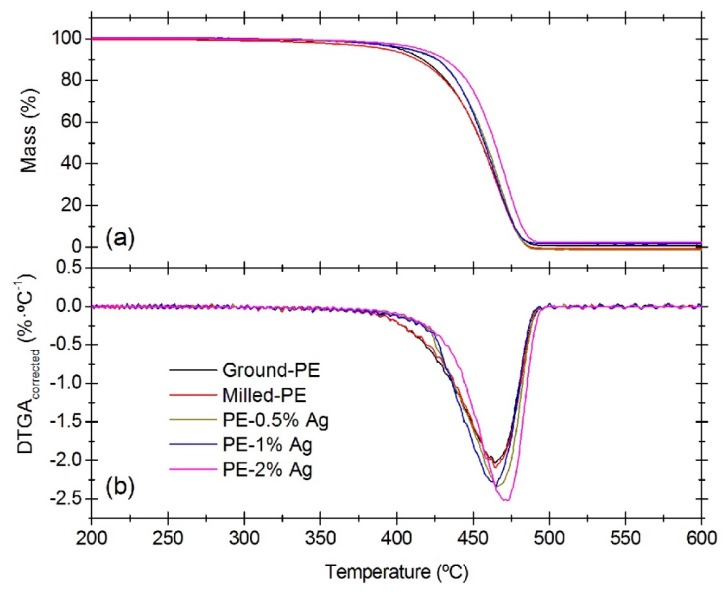
Characteristic TGA curves (**a**) and DTGA curves (**b**) of LDPE and LDPE/Ag nanocomposites.

A plot of the relative change in mass of the samples versus temperature is presented in Figure 3a, with the corresponding first derivative curve of the TGA (DTGA) in Figure 3b. The DTGA curves refer to the amount of polymer present in the sample. The characteristic transition temperatures such as *T*_5_, *T*_50_ and *T*_95_ which correspond to the temperatures for a 5%, 50% and 95% mass loss (mass %) obtained from TGA curves are listed in Table 2. The DTGA peak temperature, usually considered as a reference for thermal degradation of a substance, is also included in the table. Three different specimens of each sample were measured to obtain average values and standard deviations.

In Figure 3a it is observed that all the samples showed only one single mass-loss step representing an almost complete decomposition. No other processes, such as loss of adsorbed moisture, were detected. The plots presented in Figure 3b show that the degradation process occurs first in the samples without nanoparticles (Ground-PE and Milled-PE), and the presence of Ag NPs delayed the thermal decomposition to a certain extent. This is particularly true for the sample with 2% silver nanoparticles, PE-2% Ag. The degradation temperatures of these nanocomposites are similar to those obtained in previous studies with LDPE [38]. Comparing PE-Ground and PE-Milled, the onset of the degradation process starts at similar temperatures, which is confirmed by the data for *T*_5_ in Table 2. The values of *T*_50_ and *T*_95_ are very similar for these samples too, implying that there are no significant differences in the degradation temperature due to the milling process.

Although the data presented in Table 2 do not show significant variations for the degradation temperature at the peak (*T*_peak,DTGA_, °C), the plots in Figure 3a,b show a slight shift to higher temperatures in the degradation profile for the PE-2% Ag sample as compared to pure PE. In general, the peak associated with the main mass loss in the DTGA curve is usually sharper and has lower values for the samples containing silver nanoparticles, indicating that the mass loss at this temperature is higher. According to Figure 3b, the loss of mass during heating for the ground and milled PE samples begins before the samples with Ag NPs, but as the temperature increases, the degradation rate is higher for PE with Ag NPs.

### 2.3. Contact Angle Measurements

In general, the hydrophobic or hydrophilic character of the surface of a material significantly affects the dynamics and structure of biofilm formation [39]. The hydrophobicity or hydrophilicity of the bacterial cell is another important factor, too. If a cell surface shows a dominant hydrophobic nature, it will be more likely to wet nonpolar surfaces [39]. For example, according to a recent study [40] uniform, flat, and thin biofilms of *Pseudomonas putida* were rapidly formed on the hydrophobic surface of polyvinylidenefluoride (PVDF). In contrast, the hydrophilic surface of polyvinyl alcohol (PVA) prevented bacterial adhesion and biofilm formation; however, localized rough and thick biofilms were formed at 200 h of long-term incubation [40].

Other works have revealed that hydrophobicity can be related to surface roughness, probably being the main cause of changes in bacterial adhesion [41]. Recent research on the adhesion of *Streptococcus mutants* on modified PVDF polymer, showed that adhesion was more dependent on the specific interactions with the surface polar groups of the material than on changes in its topography [42]. For this reason, it is important then to control the properties of the surface, and therefore hot-pressed materials may inherently reduce the effect of roughness in bacterial adhesion.

Besides, a physicochemical characterization of the surface of the materials provided additional surface energy information. Contact angle measurements using the sessile drop method and surface free energy calculations were determined. Results of mean contact angles for the materials under study for each of the selected solvents (water, glycerol and diiodomethane) are collected in Table 3.

For all the liquids tested, little variations in the contact angle were observed with increasing content of Ag NPs, and most of them lie within the experimental error. Only in the case of water as the test liquid, very small changes were observed. A slight variation occurs after milling, which leads to a small decrease in the contact angle. After that, it seems that an increase in the amount of Ag NPs confers more hydrophobicity to the materials, since the contact angle increased. Analyzing the results for the different solvents, similar contact angles were observed for water and glycerol. Comparing diiodomethane to both water and glycerol, a decrease of 20° in the contact angle was observed, but there were no significant changes in the contact angle as a function of Ag NP content.

From the values of contact angles combined with the van Oss method, the values of surface free energy were calculated (See Electronic Supplementary material, Table S2). Because contact angles and surface free energy results for all Ag NP compositions were similar, it is reasonable to conclude that the presence of silver particles did not significantly affect the surface properties of the materials. Since most particles are likely to be inside the material, i.e. in the bulk, rather than on the surface, it is reasonable to surmise that the LDPE surface properties remained almost constant.

### 2.4. Bacterial Cultures

#### 2.4.1. Kirby-Bauer Diffusion Test

**Figure 4:**
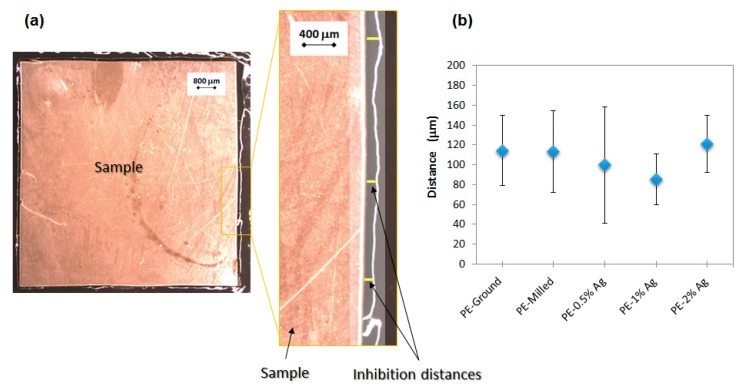
(**a**) Optical microscopy images illustrating how inhibition distances were calculated and (**b**) the results of average inhibition distances obtained for each material.

Optical micrographs were taken at different magnifications (1×, 2.5× and 4×) to measure inhibition distances along the perimeter of all samples. In Figure 4a, the micrographs at 1× (left) and 4× (zoomed area) are shown to illustrate how the inhibition distances were measured. The yellow segments highlighted in the micrograph at 4× indicate some individual measurements of this region. The size of the interphase, where no culture media grows, is considered as the inhibition zone. as bacteria did not grow here, probably due to the action of Ag NPs. Average inhibition distances of close to 120 μm were obtained for all the samples (Figure 4b) without a correlation to Ag NP content, meaning that the antimicrobial action of the particles occurs mainly on the surface of the material i.e., the action of Ag NPs is not effective outside the limits of the LDPE/Ag nanocomposites themselves. Therefore, to test the effectiveness of these materials against bacterial growth, additional experiments of biofilm development on the surface of our materials were carried out.

#### 2.4.2. Study of Biofilm Development and Bacterial Growth on the Surface of the Materials

**Figure 5:**
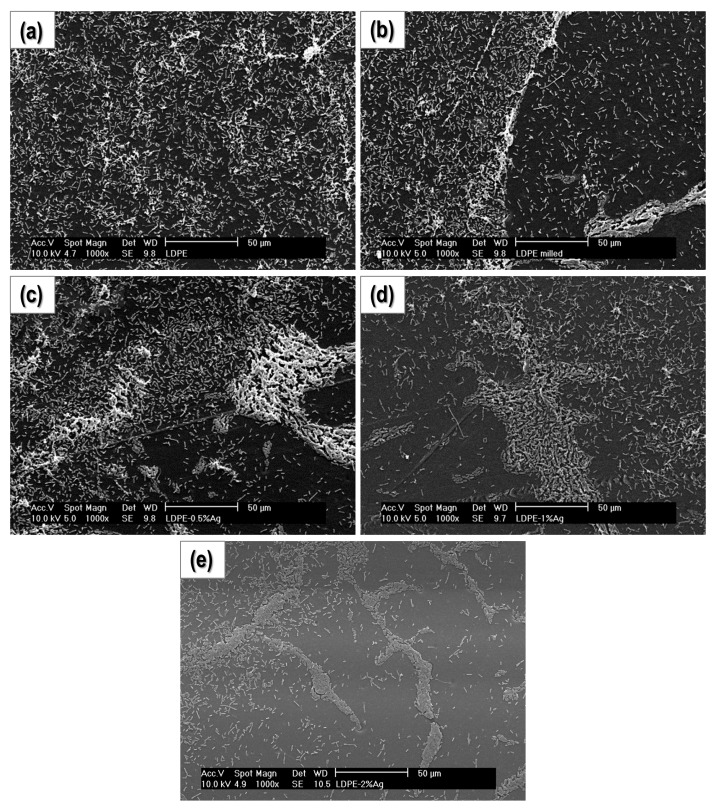
SEM micrographs obtained at 1000× for: (**a**) PE-Ground; (**b**) PE-Milled; (**c**) PE-0.5% Ag; (**d**) PE-1% Ag and (**e**) PE-2% Ag.

**Figure 6:**
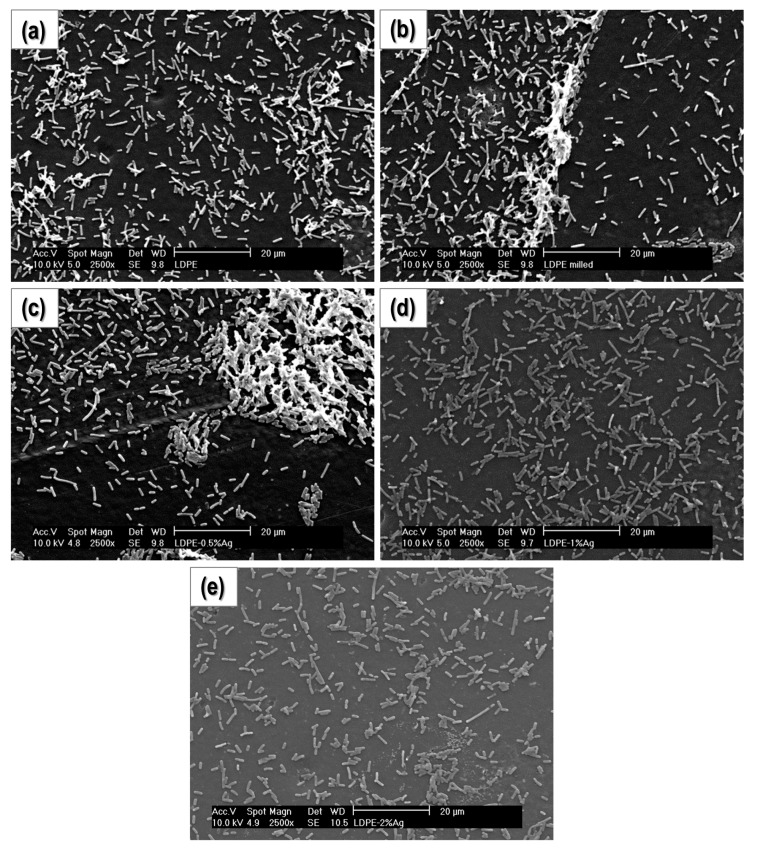
SEM micrographs obtained at 2500× for: (**a**) PE-Ground; (**b**) PE-Milled; (**c**) PE-0.5% Ag; (**d**) PE-1% Ag and (**e**) PE-2% Ag.

**Figure 7:**
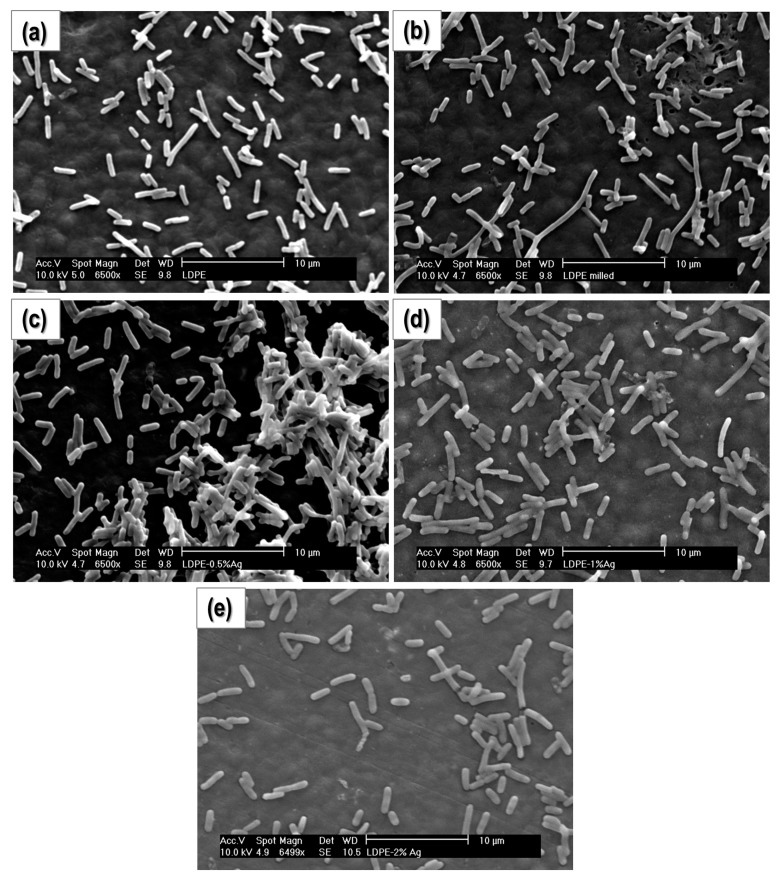
SEM micrographs obtained at 6500× for: (**a**) PE-Ground; (**b**) PE-Milled; (**c**) PE-0.5% Ag; (**d**) PE-1% Ag and (**e**) PE-2% Ag.

In these experiments, bacterial cultures were grown on the surface of the composites. After culturing, the samples were gently washed and the bacteria attached to their surfaces were fixed to avoid changes over time and improve its visualization in the microscope. In Figure 5 and Figure 6, SEM micrographs of the surfaces of the samples at 1000× and 2500×, respectively, are presented. Clearly, the concentration of bacteria on the surface of PE-2% Ag is significantly less than the other samples, and the number of bacteria appears to decrease with increasing Ag content. Figure 7 shows the same samples at a higher magnification (6500×). Again, the number of bacteria per surface unit area decreases with increasing Ag NP content. In addition, visualization of the surface of the samples for PE-milled and PE-2% Ag was done using atomic force microscopy (AFM) (See Figure S1, supplementary information). The height images revealed that the surface of polyethylene is cleaner when Ag NPs are present. The material observed on the surface may be attributed to extracellular polymeric substances (EPS), commonly attributed to polysaccharide and proteins secreted by the cells or accumulated in the extracellular space and usually present in biofilm formation and development. Therefore, not only is there a slight decrease in the amount of microorganisms but also there is less EPS, which may indicate that there is less activity.

**Figure 8:**
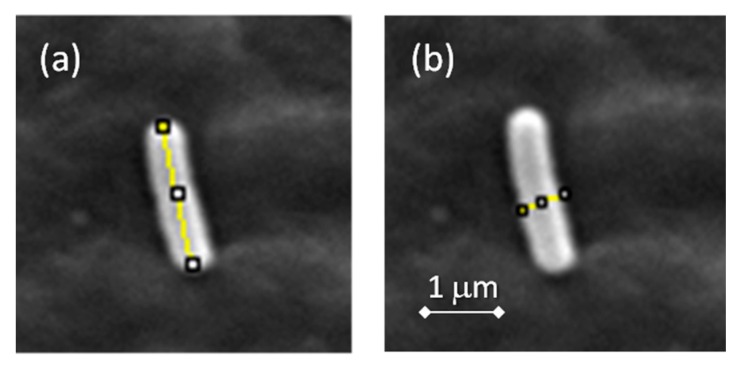
Examples of the measurements of (**a**) length and (**b**) width of a bacterium.

**Figure 9:**
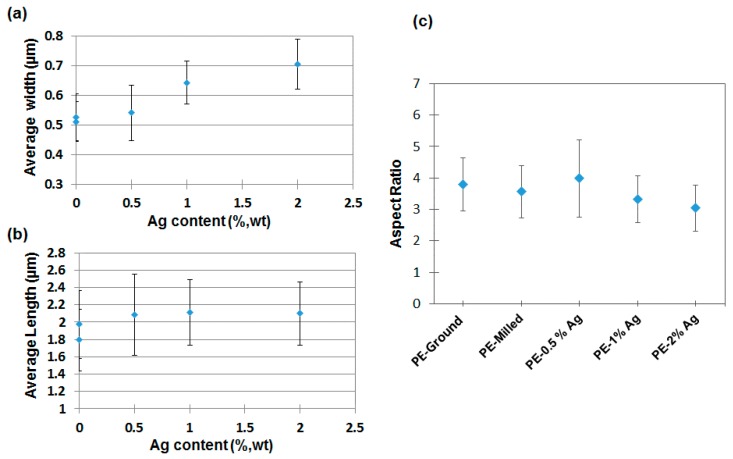
Morphological parameters of the bacteria: (**a**) Average width (μm); (**b**) average length (μm) and (**c**) aspect ratio (length/width) as a function of the content of silver particles.

Apart from the micrographs showing the quantity of bacteria that develop on the surface of the materials, the length and width of 20 bacteria on each micrograph were measured. Figure 8 illustrates how the measurements of the length and width of an isolated bacterium were done with the software ImageJ. Results of the width, length and aspect ratio (calculated as length/width) are presented in Figure 9. Average widths (Figure 9a) of the bacteria seem to increase slightly when the content of Ag NPs increases, whereas average lengths (Figure 9b) remain practically constant. Altogether, this leads to a small but significant decrease in the aspect ratio of the bacteria with the increasing Ag NP content (Figure 9c).

From this study, the presence of the Ag NPs in the PE seems to induce some changes in the morphology of the bacteria. In fact, the dimensions of the width increase significantly with increasing Ag NP content. However, this enlargement does not seem to be caused by changes in surface properties of the materials, as very little variation in contact angle measurements were observed. In a recent study, Prabhu et al. [43] suggested that Ag NPs are capable to anchor to the bacterial wall and subsequently penetrate it, thus inducing structural changes in the cell membrane. However, taking into account the materials under study this explanation is rather unlikely because it does not seem feasible that silver nanoparticles may migrate from a polymer matrix into the cell. According to a recent publication on the potential migration of silver nanoparticles from polymeric materials in contact with food, it was reported that the possibility of migration of silver nanoparticles from the polymer matrix into the food was minimal, and only particles with diameters less than 5 nm may migrate [44]. In another work, M. Carbone et al. [45] reported migration time spans of 7–10 days for Ag nanoparticles to food. Therefore, the origin of these slight changes in the cell dimensions is still unclear.

## 3. Materials and Methods

### 3.1. Materials

**Figure 10:**
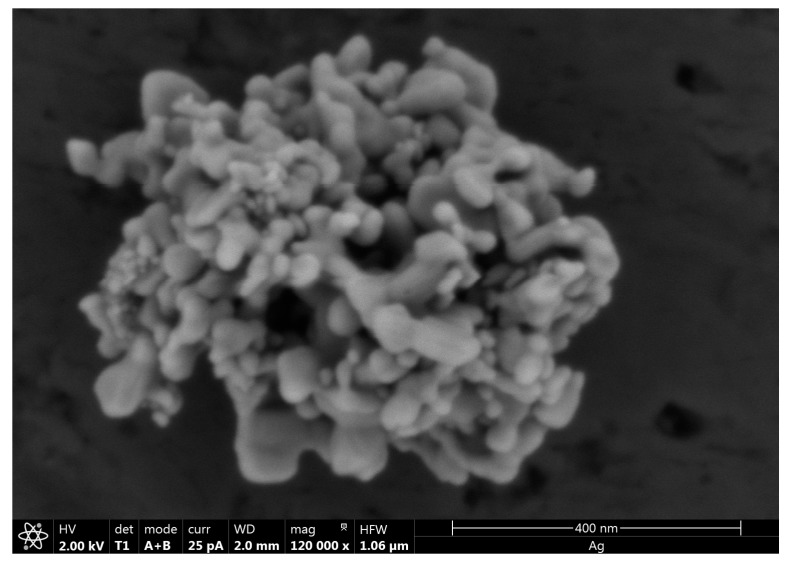
SEM micrograph of a representative aggregate of the ‘as received’ silver nanoparticles.

Polymer composites were prepared by mixing LDPE (Sigma Aldrich, San Luis, MO, USA) with commercial silver nanoparticles (HWNANO Materials, Hongwu International Group Ltd., Guangzhou, Guangdong, China), average diameter 50 nm, spherical). The XRD pattern of the commercial nanoparticles showed diffraction peaks at 2*θ* = 38.2°, 44.4°, 64.6°, 77.5° and 81.7°, which can be indexed to (111), (200), (220), (311) and (222) planes of pure silver (compared to standard powder diffraction card of Joint Committee on Powder Diffraction Standards (JCPDS) (silver file No. 04-0783). Particle size was estimated by using the Debye-Scherrer equation [46,47], resulting in mean diameter of 24 nm. In Figure 10, a micrograph obtained by scanning electron microscopy SEM (FEI’s Teneo SEM, FEI Europe Ltd., Eindhoven, The Netherlands) is shown to illustrate the presence of aggregates of approximately 400–500 nm of the ‘as received’ silver nanoparticles.

### 3.2. Sample Preparation

Sample preparation was done in two steps: (i) high energy ball milling under cryogenic conditions to disperse the particles in the polymer; and (ii) hot pressing to obtain films of the materials. A RESTCH MM400 mixer mill was used to mix the polymer with the nanoparticles. The weight percentage of Ag NPs in the composites was 0, 0.5, 1 and 2 wt %. The polymer and nanoparticle sample plus 15 stainless steel balls of 9 mm diameter were introduced into the chamber of a 50 cm^3^ stainless steel vessel, leaving one-third of the volume unoccupied to ensure optimal mixing. The samples were mixed at 25 Hz using cycles of 5 min, which was alternated with 15 min of immersion in liquid nitrogen, and the process was continuously repeated to attain 1 h of active milling.

The powders obtained after mixing (see Figure S2, Supplementary Material) were then hot pressed in a Fontijne Presses TP 400 machine (Fontijne Presses, Barendrecht, The Netherlands) to obtain films of 10 × 10 cm^2^ (see Figure S3, Supplementary Material). A sample with ground polyethylene (not milled) was also prepared as a reference material. The samples were processed at 70 kN at 140 °C for 40 min. This cycle was repeated twice and the materials obtained were cut into four pieces and re-pressed at the same conditions to obtain more homogeneous films. The surface of the materials was smooth and no porosity was observed after careful examination by SEM. The average thickness of the films processed was 130 ± 15 μm.

### 3.3. Characterization Techniques

A scanning electron microscope, FEI’s Teneo SEM (FEI Europe Ltd., Eindhoven, The Netherlands) was used. In all cases, samples were placed on the sample holders with double-sided conductive sticky tape and then subjected to sputtering with gold for 45 s, making them conductive and avoiding ‘charging artifacts’ during SEM examination. For examining biofilms formed on the surface of the materials, a Philips XL30 SEM instrument (‎FEI Europe Ltd., Eindhoven, The Netherlands) was used. In this case, micrographs at different magnifications (50×, 1000×, 2500× and 6500×) were collected. The voltage was set at 10 kV and the working distance at 10 mm.

Thermal characterization of the materials was done using differential scanning calorimetry (DSC) and thermogravimetric analysis (TGA). DSC experiments were carried out in a Mettler Toledo DSC822^e^ instrument (Greifensee, Switzerland) under nitrogen atmosphere using samples of ~2 mg. The thermal cycle was: (i) heating scan from 35 to 180 °C at 20 °C·min^−1^; (ii) cooling scan from 180 to 35 °C at 20 °C·min^−1^ and (iii) a final heating scan from 35 to 180 °C at 10 °C·min^−1^. Melting and crystallization temperatures were determined from the heating and cooling scans, respectively. Thermal degradation of the samples was studied by TGA. The measurements were carried out in a TGA-SDTA 851 Mettler Toledo thermobalance (Greifensee, Switzerland). Heating ramps from 30 °C to 750 °C at 10 °C min^−1^ were carried out under a nitrogen atmosphere with a gas flow of 20 mL·min^−1^.

Contact angles of the five samples were measured to study possible changes in surface properties of the samples due to processing conditions and to the presence of Ag NPs in the LDPE. The experiments were done using an OCA 15 Plus device from KRÜSS GmbH (Hamburg, Germany). Test liquids selected were water (dispersive, acidic and basic components), glycerol (dispersive and basic components) and diiodomethane (only dispersive component). Twenty droplet measurements were performed for each of the three liquids and samples.

### 3.4. Bacterial Cultures

Two approaches to test the behavior of the materials against bacterial growth were considered. First, to study the possible antibacterial action of the materials as a function of the distance to the surface of the material, a modification of the traditional Kirby-Bauer test was done (See Figure S4 supplementary material). Gram negative *E. coli*, DH5α Competent cells, from ThermoFischer Scientific (Waltham, MA, USA) were used. The frozen bacteria were thawed. A solution of 90 μL of bacteria in 910 μL of Luria Bertani (LB) media was incubated at 37 °C for 30 min. From this solution, 200 μL were introduced as seeds in an LB agar plate. Then, squares of ~1 cm^2^ of the samples were placed in the agar plate and incubated at 37 °C overnight. After that, with an Olympus optical microscope, images of the incubated samples were captured at different magnifications (1×, 2.5× and 4×). The inhibition distances were calculated using image analysis software (analySIS getIT, Olympus, Tokyo, Japan).

In the second approach, cultures to study biofilm formation on the surface of the materials were grown in a 24-microwell plate (ThermoFischer Scientific) using the same *E. coli* strain. For this experiment, squared samples of the films (0.8 × 0.8 cm^2^) were cut and glued with an epoxy adhesive (92 NURAL, Henkel, Düsseldorf, Germany) onto stainless steel sample plates (10 mm diameter). Prior to incubation, all the samples were sterilized by spraying on a 70% solution of ethanol and then dried in a sterile laminar flow hood. From this point on, all the processes were carried out in a sterile environment. Over the sterilized samples, 1 mL of the previously prepared 1/100 dilution was added to each well and incubated for 3 h at 37 °C. After incubation, the films were gently washed and rinsed with 1 mL of saline solution (NaCl 0.9 wt %) to remove poorly attached bacteria, leaving only bacteria adhered to the surface of the materials as a biofilm.

For SEM visualization, the samples were fixed by adding 1 mL of 2.5 wt % glutaraldehyde to each well. After 30 min, the glutaraldehyde solution was removed and samples were rinsed 3 times with a phosphate buffered solution (PBS) to remove remaining glutaraldehyde. Once fixation was completed, the samples were dehydrated by immersing them in solutions of increasing (30, 50, 70 and 100%) ethanol concentrations for 10 min in each case. Finally, the ethanol was removed and samples were left in the laminar flow hood until fully dry.

## 4. Conclusions

Composites based on LDPE and Ag NPs were prepared using HEBM followed by hot-pressing to obtain films. The novelty of this work relies on the use of a facile processing method that may be easily transferred to industry to produce nanocomposite materials commercially. Although mixing and milling were used to maximize homogeneity, Ag aggregates of ~400–500 nm were observed. The presence of the silver particles did not modify either the thermal properties or the surface properties of the materials. A Kirby-Bauer diffusion test and SEM examination of the biofilm development on the surface of samples revealed that the introduction of Ag NPs is effective against bacterial growth on the composite surfaces. Therefore, these materials may find potential applications in the food and health industries, particularly antibacterial storage materials or general-purpose containers. Finally, image analysis on the aspect ratio of bacteria revealed a slight decrease in the aspect ratio of the bacteria. These changes are related to the presence of Ag NPs, but further studies are needed to understand the mechanism.

## Figures and Tables

**Table 1:** Melting temperatures obtained from the first heating (*T*_m,1_) and second heating (*T*_m,2_) scan, crystallization temperature (*T*_c_) and crystallization degree (*X*_c_).

Sample	*T*_m,1_ (°C) (1st Scan)	*T*_m,2_ (°C) (2nd Scan)	*T*_c_ (°C) (Cooling)	*X*_c_
PE-Ground	112.6	109.8	96.7	0.30
PE-Milled	111.3	109.7	98.5	0.29
PE-0.5% Ag	110.0	109.7	98.5	0.29
PE-1% Ag	110.7	110.2	98.4	0.30
PE-2% Ag	111.3	110.5	98.1	0.29

**Table 2:** Degradation temperatures obtained from TGA and DTGA curves and residual mass for each sample (*T*_5_, *T*_50_ and *T*_95_ correspond to 5%, 50% and 95% mass loss).

Sample	*T*_5_ (°C)	*T*_50_ (°C)	*T*_95_ (°C)	*T*_peak,DTGA_ (°C)	Residue (Mass %)
PE-Ground	403 ± 2	456 ± 2	481 ± 1	466 ± 0	0.0
PE-Milled	395 ± 6	456 ± 1	484 ± 1	465 ± 0	0.0
PE-0.5% Ag	408 ± 6	458 ± 4	481 ± 2	467 ± 3	0.7
PE-1% Ag	406 ± 2	459 ± 2	483 ± 2	466 ± 1	1.9
PE-2% Ag	410 ± 8	463 ± 1	486 ± 1	470 ± 3	2.2

**Table 3:** Average values of contact angle for the samples in water, glycerol and diiodomethane.

Sample	Water	Glycerol	Diiodomethane
PE-Ground	101 ± 2	95 ± 2	74 ± 1
PE-Milled	95 ± 2	94 ± 1	73 ± 2
PE-0.5% Ag	93 ± 2	96 ± 1	72 ± 3
PE-1% Ag	94 ± 1	93 ± 2	73 ± 2
PE-2% Ag	97 ± 1	95 ± 1	72 ± 2

## Supplementary Materials

The following are available online at http://www.mdpi.com/2079-4991/8/2/60/s1, Figure S1: Height AFM images obtained for: (a) PE-milled and (b) PE-2%Ag NPs, Figure S2: Example of the powders of milled materials with different content in AgNPs: (a) PE Grinded; (b) PE milled; (c) PE-0.5% Ag; (d) PE-1%Ag and (e) PE-2%Ag. (PE Grinded- not milled- was studied as reference material), Figure S3: Pressure-temperature ramp used to prepare the films of the materials; (b) Example of the 10 cm × 10 cm materials obtained after the hot pressing cycle, Figure S4: Experimental set-up used for the Kirby-Bauer diffusion test. Table S1: Melting enthalpies associated to the first heating (Tm,1) and second heating (Tm,2) scan, crystallization temperature (Tc) and crystallization degree (Xc), Table S2: Surface energy values (mN/m) and its main components (dispersive, acidic and basic, also in mN/m) as calculated with the van Oss method.
