# Supplementary material for: Preparation and Characterization of Antimicrobial Films Based on LDPE/Ag Nanoparticles with Potential Uses in Food and Health Industries
Source: Nanomaterials (Basel). 2018 Jan 24;8(2):60. doi: 10.3390/nano8020060 (PMC5853693; doi:10.3390/nano8020060)
Supplement: Supplementary file 1 [file nanomaterials-08-00060-s001.pdf]

nanomaterials

MDPI

Article

Preparation and Characterization of Antimicrobial Films Based on LDPE/Ag Nanoparticles with Potential Uses in Food and Health Industries

Dania Olmos [1,*], Gloria María Pontes-Quero [1], Angélica Corral [2] Gustavo González-Gaitano [3] and Javier González-Benito [1,*]

[1] Department of Materials Science and Engineering, Instituto de Química y Materiales Álvaro Alonso Barba (IQMAA), Universidad Carlos III de Madrid, Leganés 28911, Madrid, Spain; gpontes@ing.uc3m.es
[2] Department of Bioengineering and Aerospace Engineering, TERMeG, Universidad Carlos III de Madrid, Leganés 28911, Madrid, Spain; angelica.corral@uc3m.es
[3] Department of Chemistry, Facultad de Ciencias, Universidad de Navarra; Pamplona 31080, Spain; gaitano@unav.es
* Correspondence: dolmos@ing.uc3m.es (D.O.); javid@ing.uc3m.es (J.G.-B.); Tel.: +34-91-624-9447 (D.O.); +34-91-624-8870 (J.G.-B.)

Differential scanning calorimetry (DSC)

Table S1. Melting enthalpies associated to the first heating ($T_{m,1}$) and second heating ($T_{m,2}$) scan, crystallization temperature (T_c) and crystallization degree (χ_c).

Sample	$\Delta H_{m,1}$ (J·g^{-1})	$\Delta H_{m,2}$ (J·g^{-1})	ΔH_c (J·g^{-1})	χ_c
**PE-GROUND**	84.64	89.97	-85.07	0.30
**PE- MILLED**	91.15	88.27	-81.02	0.29
**PE- 0.5% Ag**	87.8	87.08	-79.94	0.29
**PE-1% Ag**	82.07	91.45	-81.99	0.30
**PE-2% Ag**	84.18	86.77	-80.54	0.29

To calculate the degree of crystallization, X_c, the following equation was used (eq. 1).

$$X_i = \frac{\Delta H_m}{(1-x)\cdot\Delta H_m^0} \quad (1)$$

where x is the weight fraction of particles and $\Delta H_m°$ is the standard enthalpy of fusion for the fully crystallized polyethylene, $\Delta H_m°$= 289.9 J/g [37].

Contact angle measurements

Surface energies according to the van Oss method were calculated with the aid of the software. Results of the surface energy of the materials and its main components are shown in Table S2.

Table S2. Surface energy values (mN/m) and its main components (dispersive, acidic and basic, also in mN/m) as calculated with the van Oss method.

Sample	Surface energy (mN/m)	Dispersive component (mN/m)	Acidic component (mN/m)	Basic component (mN/m)
**PE-GROUND**	21 ± 2	21 ± 1	0 ± 0	3 ± 2
**PE- MILLED**	20 ± 2	20 ± 1	0 ± 0	5 ± 2
**PE- 0.5% Ag**	18 ± 2	18 ± 2	0 ± 0	7 ± 2

**PE-1% Ag**	21 ± 2	21 ± 1	0 ± 0	6 ± 2
**PE-2% Ag**	21 ± 2	21 ± 1	0 ± 0	4 ± 1

Visualization of the surface of PE and PE-2% Ag with Atomic Force Microscopy

Morphological characterization of the films was done using a scanning probe microscope MultiMode Nanoscope IVA (from Veeco, now Bruker, MA, USA), AFM, at ambient conditions in tapping mode with etched silicon probes (40 N/m).

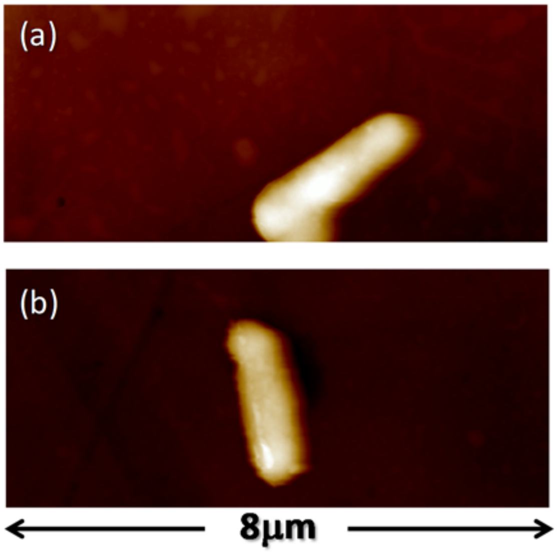

Figure S1. Height AFM images obtained for: (**a**) PE-milled and (**b**) PE-2% Ag NPs.

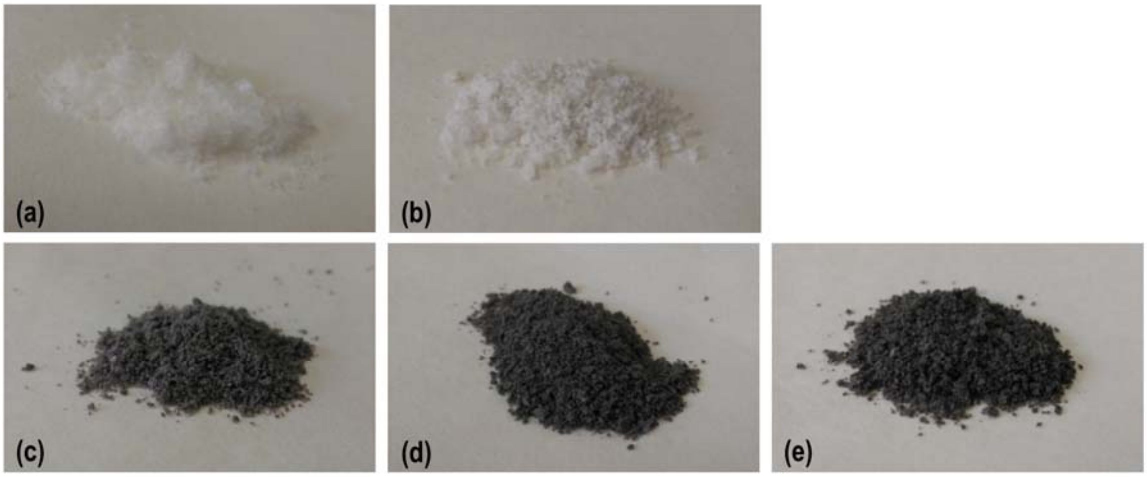

Figure S2. Example of the powders of milled materials with different content in AgNPs: (**a**) PE Grinded; (**b**) PE milled; (**c**) PE-0.5% Ag; (**d**) PE-1%Ag and (**e**) PE-2%Ag. (PE Grinded- not milled- was studied as reference material).

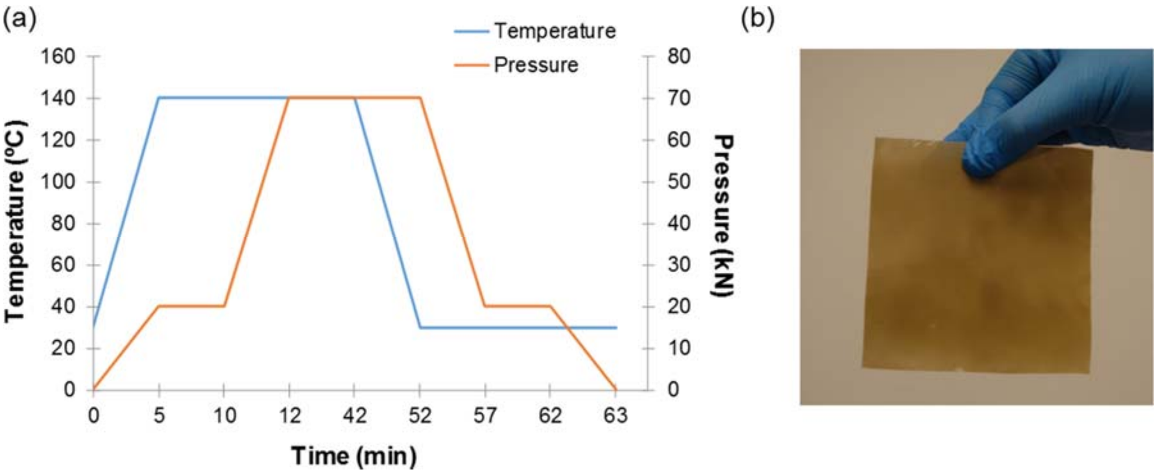

Figure S3. (**a**) Pressure-temperature ramp used to prepare the films of the materials; (**b**) Example of the 10 cm × 10 cm materials obtained after the hot pressing cycle.

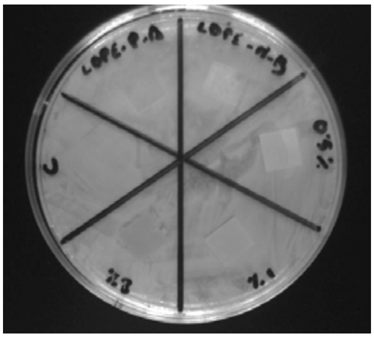

Figure S4. Experimental set-up used for the Kirby-Bauer diffusion test.

Calculations of particle size of commercial Ag NPs

Particle size was estimated by using the Debye-Sherrer's equation (equation (2) from references [46,47]):

$$D = \frac{0.9 \times \lambda}{\beta \times \cos\theta} = \frac{0.9 \times 0.1541}{\left(0.42 \times \frac{\pi}{180}\right) \times 0.9449} \quad (2)$$

where λ is wave length of X-ray (0.1541 nm), β is the width at half maximum in radians, FWHM (full width height maximum), θ is the diffraction angle and D is particle diameter size. Making calculations a mean diameter of 24.1 nm was obtained. This data refers to the crystal grain size and the commercial value of size refers to the particle size observed by microscopy which agrees well with our own results. These results can be explained considering that, probably, due to the process of nanoparticles fabrication some of them appears well joined as it is observed in the following micrograph obtained by scanning electron microscopy, SEM (Figure 10).

References (from the manuscript)

37. Mandelkern, L. *Crystallization of Polymers in Equilibrium Concepts*, 2nd ed.; Cambridge University Press: New York, NY, USA, 2002; Volume 1.
46. Scherrer, P. Bestimmung der Grösse und der inneren Struktur von Kolloidteilchen mittels Röntgenstrahlen. In *Kolloidchemie Ein Lehrbuch*; Springer: Berlin/Heidelberg, Germany, 1918; Volume 26, pp. 98–100.
47. Wilson, J.I.L. A. J. C. Scherrer after Sixty Years: A Survey and Some New Results in the Determination of Crystallite Size. *J. Appl. Crystallogr.* **1978**, *11*, 102–113.
